# Deep learning-based classification of erosion, synovitis and osteitis in hand MRI of patients with inflammatory arthritis

**DOI:** 10.1136/rmdopen-2024-004273

**Published:** 2024-06-17

**Authors:** Maja Schlereth, Melek Yalcin Mutlu, Jonas Utz, Sara Bayat, Tobias Heimann, Jingna Qiu, Chris Ehring, Chang Liu, Michael Uder, Arnd Kleyer, David Simon, Frank Roemer, Georg Schett, Katharina Breininger, Filippo Fagni

**Affiliations:** 1 Department of Artificial Intelligence in Biomedical Engineering, Friedrich-Alexander-Universität Erlangen-Nürnberg, Erlangen, Germany; 2 Department of Internal Medicine 3—Rheumatology and Immunology, Friedrich-Alexander-University Erlangen-Nürnberg (FAU) and Universitätsklinikum Erlangen, Erlangen, Germany; 3 Deutsches Zentrum Immuntherapie (DZI), Friedrich-Alexander-University Erlangen-Nürnberg (FAU) and Universitätsklinikum Erlangen, Erlangen, Germany; 4 Digital Technology and Innovation, Siemens Healthcare GmbH, Erlangen, Germany; 5 Institute of Radiology, Friedrich-Alexander-University Erlangen-Nürnberg (FAU) and Universitätsklinikum Erlangen, Erlangen, Germany; 6 Department of Computer Science, Friedrich-Alexander-Universität Erlangen-Nürnberg, Erlangen, Germany; 7 Department of Rheumatology and Clinical Immunology, Charité - Universitätsmedizin Berlin, Berlin, Germany; 8 Department of Radiology, Boston University School of Medicine, Boston, Massachusetts, USA

**Keywords:** rheumatoid arthritis, psoriatic arthritis, machine learning, magnetic resonance imaging, classification

## Abstract

**Objectives:**

To train, test and validate the performance of a convolutional neural network (CNN)-based approach for the automated assessment of bone erosions, osteitis and synovitis in hand MRI of patients with inflammatory arthritis.

**Methods:**

Hand MRIs (coronal T1-weighted, T2-weighted fat-suppressed, T1-weighted fat-suppressed contrast-enhanced) of rheumatoid arthritis (RA) and psoriatic arthritis (PsA) patients from the rheumatology department of the Erlangen University Hospital were assessed by two expert rheumatologists using the Outcome Measures in Rheumatology-validated RA MRI Scoring System and PsA MRI Scoring System scores and were used to train, validate and test CNNs to automatically score erosions, osteitis and synovitis. Scoring performance was compared with human annotations in terms of macro-area under the receiver operating characteristic curve (AUC) and balanced accuracy using fivefold cross-validation. Validation was performed on an independent dataset of MRIs from a second patient cohort.

**Results:**

In total, 211 MRIs from 112 patients (14 906 region of interests (ROIs)) were included for training/internal validation using cross-validation and 220 MRIs from 75 patients (11 040 ROIs) for external validation of the networks. The networks achieved high mean (SD) macro-AUC of 92%±1% for erosions, 91%±2% for osteitis and 85%±2% for synovitis. Compared with human annotation, CNNs achieved a high mean Spearman correlation for erosions (90±2%), osteitis (78±8%) and synovitis (69±7%), which remained consistent in the validation dataset.

**Conclusions:**

We developed a CNN-based automated scoring system that allowed a rapid grading of erosions, osteitis and synovitis with good diagnostic accuracy and using less MRI sequences compared with conventional scoring. This CNN-based approach may help develop standardised cost-efficient and time-efficient assessments of hand MRIs for patients with arthritis.

**WHAT IS ALREADY KNOWN ON THIS TOPIC**

- Assessing and scoring MRI is a costly and time-consuming process that still limits the use of MRI in clinical trials and practice.
- Artificial intelligence (AI) approaches have already been implemented in rheumatological imaging to assist the interpretation of X-rays and ultrasound images of osteoarthritis and arthritis. However, no AI tool for the in-depth scoring of arthritis on hand MRI has yet been developed.

**WHAT THIS STUDY ADDS**

- We developed and validated a convolutional neural network-based automated scoring system that allowed the automatic detection and grading of erosions, osteitis and synovitis in hand MRI of patients with RA and PsA.
- Our system achieved good diagnostic accuracy using less MRI sequences compared with conventional scoring. In particular, sequences without contrast enhancement performed comparably to contrast-enhanced sequences for the detection of erosions.
- Translating the use of our system to clinical use such as in the setting of a clinical trial requires further steps, including training it with larger cohorts from multiple centres, validating the algorithm’s performance on longitudinal follow-up data, as well as automating the process of landmark detection.

**HOW THIS STUDY MIGHT AFFECT RESEARCH, PRACTICE OR POLICY**

- This AI-based approach to MRI may help develop standardised cost-efficient and time-efficient tools to assist the assessment of hand MRI scans for patients with arthritis. If successfully translated into practice, this could help expand the use of MRI and optimising clinical decision-making.

## Introduction

Inflammatory arthritides, such as rheumatoid arthritis (RA) and psoriatic arthritis (PsA), are chronic progressive immune-mediated diseases characterised by extensive joint destruction leading to chronic pain, disability and increased morbidity if left untreated.^1 2^ The hand and wrist joints are the most prominently involved anatomical regions in inflammatory arthritis in terms of frequency as well as prognostic significance.^3–5^ Lesions of the carpal and finger joints and of the adjacent structures, such as synovitis, tenosynovitis and osteitis, are frequent and are associated with pain, swelling, impaired hand function^6–8^ and lead to an increased risk of developing bone erosions.^9^ The presence of erosions both in RA and PsA is a major prognostic factor for disease outcomes and is associated with disability, reduced response to therapies and increased all-cause mortality.^10^ Following, the occurrence of new hand bone erosions is the most frequently used outcome measure for imaging in RA and PsA clinical trials.^11^ Among the available imaging techniques, MRI is the only method that allows a comprehensive three-dimensional (3-D) assessment of both bone and soft tissues, enabling the visualisation of early signs of inflammation as well as structural changes.^12^ The RA MRI Scoring Systems (RAMRIS) and the PsA MRI Scoring System (PSAMRIS) are Outcome Measures in Rheumatology (OMERACT)-endorsed MRI scores, which are used to assess and quantify pathological changes of the hand in RA and PsA, respectively. However, despite reports of high agreement between raters,^13 14^ performing such scorings is a costly and time-consuming process that requires interpretation by an experienced rheumatologist or radiologist, hence greatly limiting their use.

To overcome some of these challenges, there is growing interest in developing artificial intelligence (AI)-based automated methods for detecting structural pathology in MRI images. Machine learning approaches have already been successfully implemented in rheumatological imaging such as ultrasound^15^ and X-ray,^16 17^ but only a limited number of studies have investigated machine learning applications for hand MRIs, such as for the classification of arthritis^18^ and for predicting the development of arthritis in at-risk patients,^19^ yielding encouraging results. To the best of our knowledge, no machine learning tool has yet been developed for the recognition and quantification of specific pathological changes in hand MRI images of arthritis patients. Automated detection systems hold the promise of providing fast, objective and standardised assessments for use in clinical trials as well as in routine clinical practice to monitor disease activity and response to therapies.^20^

In this study, we present a novel machine learning-based approach to automatically score erosions, synovitis and osteitis in MRI images of RA and PsA patients. Moreover, the relevance of different MRI sequence types is investigated. The trained algorithm is validated on an independent validation dataset of hand MRI images.

## Methods

### Patients

Patients contributing to the training/internal validation and external validation dataset were recruited at the outpatient clinic of the department of rheumatology and immunology of the University Hospital of Erlangen between January 2010 and July 2023 and were all part of the Erlangen imaging cohort, which has been described elsewhere.^21^ Briefly, patients with a diagnosis of RA fulfilling the 2010 American College of Rheumatology/European League Against Rheumatism classification criteria^22^ and patients with PsA fulfilling the classification criteria for PsA^23^ for whom at least one hand MRI was available were considered eligible for study participation. Patients with overlapping diagnoses of other immune-mediated inflammatory diseases were excluded from study participation.

For training/internal validation, hand MRI images from three different clinical studies were selected: the BAREBONE trial (Effect of Baricitinib Treatment on Peripheral Bone in RA; single-arm, prospective, phase IV trial to evaluate the effects of baricitinib on bone properties in active RA; NCT03701789), the EPos trial (Early PsA on treatment strategy; single-arm, prospective, phase IV trial to assess the effects of apremilast on bone changes in early PsA; EUDRACT (European Union Drug Regulating Authorities Clinical Trials): 2018-000335-27) and the ABEPSA trial (Abatacept Bone Effects in Psoriatic Arthritis; single-arm, prospective, phase IV trial to assess the effects of abatacept on bone changes in PsA; NCT04106804). In order to improve the algorithm by training it with a higher number of pathological findings and increase the variability of the training dataset, we also included a subset of hand MRIs performed at our hospital as routine clinical practice in patients with high disease activity (Disease Activity Score 28 (DAS28) score>5.1). For the external validation dataset, MRI images originated from the PARAJA cohort (Prospective cohort of PsA und RA patients treated with Janus Kinase inhibitors; a prospective clinical and imaging cohort of RA and PsA patients treated with JAK inhibitors; #19_18B) and from the clinical trial PSARTROS (Psoriasis-Arthritis & Bone Program; single-arm, prospective, phase IV trial to assess the effects of secukinumab on inflammatory and structural changes of the hands in PsA; NCT02483234).

The studies included scans at different timepoints for one patient with at least 3 months between scans. Additionally, each patient was only included in either one fold during cross-validation of the training/internal validation dataset or external validation. The association of each study to either dataset was according to their availability and release, as PARAJA and PSARTROS were available at a later date. This choice can be seen as random. Finally, demographic and clinical information were collected for all patients. A flow chart of the study design is available in online supplemental figure S3.

10.1136/rmdopen-2024-004273.supp1 Supplementary data

### MRI methods

MRI scans of the hand were acquired on 1.5 T Magnetom Avanto and Aera systems (Siemens, Erlangen, Germany) for the training/internal validation and the external validation dataset. The MRI protocols were identical for both systems and consisted of five sequences: coronal T1-weighted (w) turbo spin echo (slice thickness 2 mm, repetition time (TR) 666 ms, echo time (TE) 12 ms, echo train length (ETL) 4, 384×252 matrix, flip angle 150, field of view (FOV) 158×240 mm), coronal T2w fat-suppressed (fs) (turbo inversion recovery magnitude (TIRM)—slice thickness 2 mm, TR 3500 ms, TE 60 ms, inversion time (TI) 160 ms, ETL 8, 448×358 matrix, flip angle 180, FOV 220×220 mm), axial T2 fs (slice thickness 3 mm, TR 4620 ms, TE 72 ms, ETL 12, 320×224 matrix, flip angle 180, FOV 150×150 mm), axial T1w fs contrast-enhanced (CE) (slice thickness 3 mm, TR 760 ms, TE 13 ms, ETL 3, 240×320 matrix, flip angle 150, FOV 150×150 mm) and coronal T1w fs images (slice thickness 2.5 mm, TR 571 ms, TE 11 ms, ETL 3, 512×384 matrix, flip angle 150, FOV 220×220 mm) after intravenous gadolinium injection (0.2 mL/kg) were used. Bone erosions were evaluated by using coronal T1w turbo spin echo and axial T1w CE sequences. The coronal and axial T1w fs CE images were assessed to evaluate tenosynovitis and periarticular inflammation along with axial images for confirmation of the findings on a perpendicular plane. T2w coronal and axial (fs TIRM) sequences were used to assess osteitis.

### Image scoring, preparation and annotation

**Figure 1:**
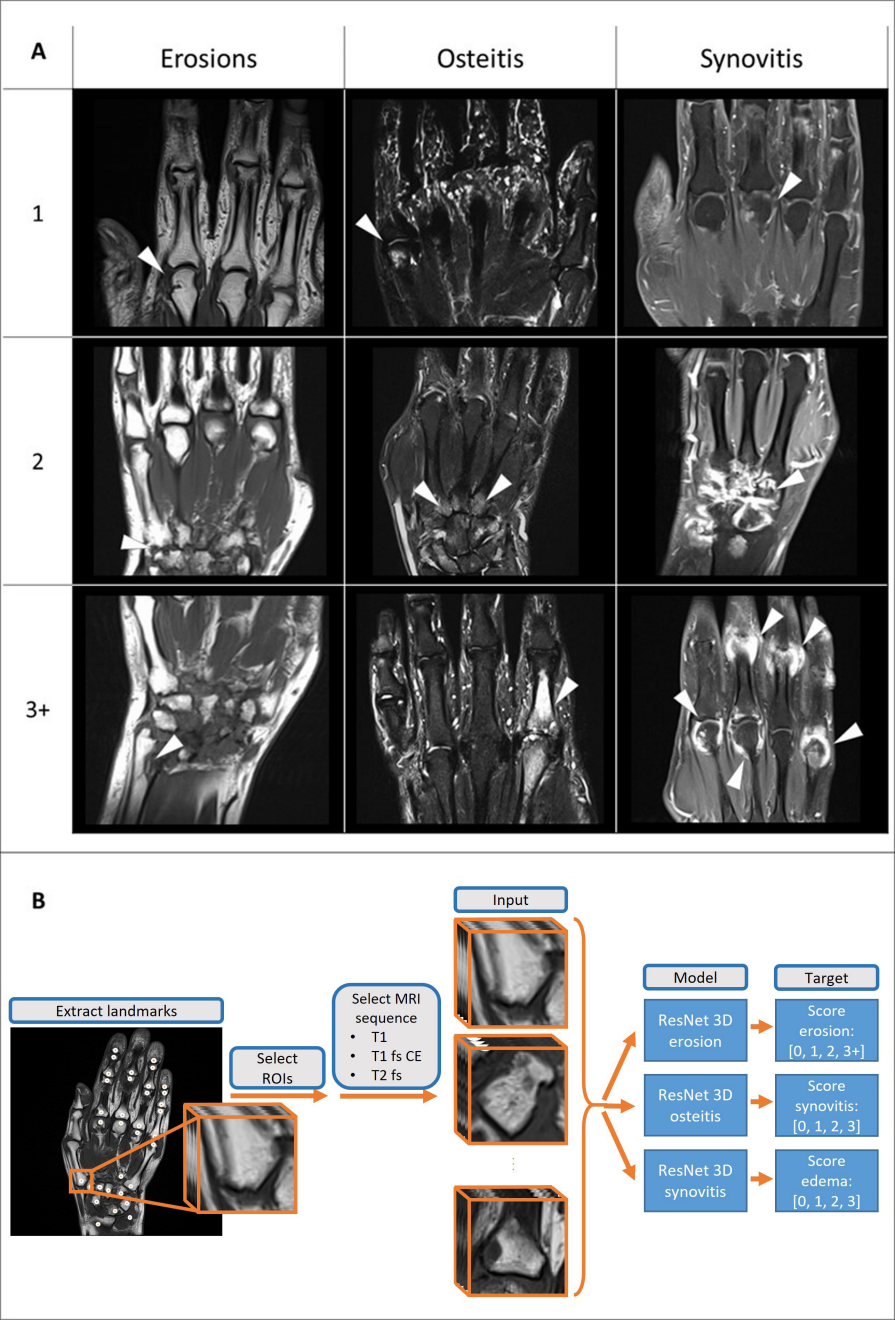
(A) MRI scans showing examples of erosions, osteitis and synovitis (arrowheads) as categorised by the neural networks based on the Rheumatoid Arthritis MRI Scoring System and Psoriatic Arthritis MRI Scoring System in the regions of interest relative to each score. (B) Neural network pipeline. Region of interests (ROIs) required for scoring of erosion, osteitis and synovitis are extracted from hand MRI images. All ROIs are fed into a ResNet 3D to detect each score, respectively. *The 3+ category was only used for erosions. Scores for osteitis and synovitis ranged from 0 to 3.

The region of interests (ROIs) included in the RAMRIS^24^ and in PsAMRIS^25^ scoring systems were used for our analysis. Detailed information on the ROIs can be found in online supplemental file 1. Each anatomical ROI was assessed manually by one expert reader (training dataset: either MYM or SB, rheumatologists with 4 years of MRI experience) blinded to all patient data except diagnosis. Agreement between the two readers was validated by a reader study for which a subset of MRIs were scored by both assessors (detailed information is reported in the dedicated paragraph; external validation dataset: MYM). Erosions, osteitis and synovitis were scored using the RAMRIS and PsAMRIS scoring systems according to OMERACT recommendations, which categorise pathologic changes on an ordinal scale for erosions (0–10), osteitis (0–3) and synovitis (0–3). As scores higher than three only occurred for erosions, we used an adapted score ranging from 0 to 3+, which included all scores≥3. Examples for each pathology and score are depicted in figure 1A.

To spatially define the ROIs, reference landmarks were manually added to each MRI image. A landmark corresponds to the 3-D centre of the anatomical ROI. Our approach used a custom landmark annotation tool. A graphical representation of this process is depicted in figure 1B. Initial experiments showed very high agreement between different annotators. For each MRI image, 23 and 24 landmarks for erosions and osteitis and 7 and 12 landmarks for synovitis were annotated for RA and PsA patients, respectively. Subsequently, 3-D ROIs were extracted automatically. For erosions and osteitis, a size of 30×30×30 mm was chosen for all ROIs except radius and ulna with 45×45×45 mm. For synovitis, all ROIs have a size of 45×45×45 mm except the radioulnar and radiocarpal ROIs with 30×30×30 mm.

### Deep learning architecture for ROI scoring

For each MRI sequence type and each pathology, a separate ResNet-3D pretrained on a video classification task^26 27^ was trained for score prediction. In initial experiments, a Swin Transformer architecture^28^ with pretrained weights on a video classification task was compared with the ResNet-3D (online supplemental table S18). As only a small number of samples for our training was available, we did not observe a performance improvement when using the transformer architecture. As the transformer has higher computational costs, we decided to use the comparable ResNet-3D architecture. For automated prediction, only coronal MRI images (T1, T1 fs CE, T2 fs) were employed. The results of the sequence-specific networks were subsequently fused using a majority voting of individual sequence predictions. Additionally, we calculate a compound score for each patient by summing all lesion within one pathology to better estimate the overall correlation of predicted and actual disease activity. ROIs were individually normalised in the range (0, 1) and reshaped to the same size of 16×128×128 voxels. During training, random augmentations including brightness increase up to 20%, contrast increase up to 30%, rotation between −10° and +10°, flipping, 90° rotation and Gaussian noise were applied with a probability of 50% each. For all experiments, fivefold cross-validation was used, which results in a data split of 80% for training and 20% for testing. To prevent data leakage, each patient was only present in either of the sets. An additional external validation on an independent patient cohort was performed. All experiments were executed on either an A100, V100 or RTX3080 GPU (NVIDIA) using PyTorch V.1.13.1 and Python V.3.10.9. Each model was trained for 250 epochs with early stopping after 20 epochs of no improvement. The AdamW optimiser with default parameters was used with a learning rate of 0.0001. Weighted subsampling was applied for all tasks to handle class imbalance. The batch size was set to 10 for erosions and osteitis and 5 for synovitis. For the loss functions, cross-entropy loss was used for erosions and osteitis, while focal loss with a gamma of 0.7 was applied for synovitis. A slightly different training scheme including loss function and batch size was applied for synovitis, as the number of training data samples available in this case was smaller.

### Impact of training data size on neural network performance

In order to gain insights into the influence of different training data sizes and into the variance within the study data, we investigated the performance of the neural network under different training set sizes in terms of area under the receiver operating characteristic curve (AUC) while keeping the test set unchanged.

### Reader study

A reader study was performed with the aim of determining the inter-rater variability between expert readers. This indicates a natural upper bound for the automatic scoring. The reader study examined the agreement of experts with regard to classification performance of erosions, osteitis and synovitis based on the systematic evaluation of the same MRI scans. The human readers (MYM and SB) were blinded to all clinical and demographic information regarding the study patients. A subset of 30 RA patients from the BAREBONE trial was selected. To match the network scores, predictions higher than or equal to three were grouped within the class 3+.

### Statistical methods

Patient characteristics were represented as summary statistics for continuous and categorical data. The selected metrics are commonly used in related work.^15 18^ The macro AUC measures how well a model distinguishes between positive and negative cases based on various threshold settings for each class separately, avoiding strong influence from the dominant class.^29 30^ The SD of the macro AUCs was calculated during a five-fold stratified cross-validation. We compare the accuracy of the classification performed by the algorithm against a reference macro AUC, which is the value expected in case of a random classification (ie, AUC≤0.5). We further computed the balanced accuracy, which calculates the average of recall and specificity for each class and compared the results against the value expected in case of a random classification of the four scores (0, 1, 2, 3+; ie, balanced accuracy≤0.25). Last, the weighted PR-AUC (Precision-Recall AUC) was calculated, which summarises the area under the precision-recall curve, that is, the trade-off between positive predictions and true positive rate depending on the class frequency. Summarising, macro AUC estimates how well the model separates classes, while balanced accuracy measures how well all classes can be predicted.

Regarding the reader study, Cohen’s kappa coefficients, balanced accuracy and macro AUC were computed to calculate the inter-rater agreement.

## Results

### Patient characteristics

A total of 77 patients with RA and 35 patients with PsA were included in the study and provided MRI data for the training dataset. 63% of participants were women, the mean (SD) age of the training cohort was 54.1 (± 12.4) years, and the mean (SD) body mass index was 28.5 (±6.1) kg/m^2^ (table 1). Disease activity was variable throughout the cohort with overall mean (IQR) TJC (tender joint count) of 2 (0–4) and SJC (swollen joint count) of 0 (0–2) and a mean (SD) DAS28 of 3.3 (1.3). Inflammatory activity on MRI was moderate and overall higher in patients with RA compared with PsA; the mean (SD) RAMRIS Score was 21.4 (3.5), while mean PsAMRIS (SD) was 7.2 (5.7). Detailed patients’ clinical characteristics and therapy as well as imaging features for each group are reported in table 1.

**Table 1:** Patient clinical and imaging characteristics of the training/internal validation dataset

N	Total	RA	PsA
	112	77	35
Demographics			
Female sex, n (%)	71 (63.4)	56 (72.7)	15 (42.9)
Age (years), mean (SD)	54.1 (12.4)	53.1 (11.3)	43.7 (18.5)
BMI (kg/m^2^), mean (SD)	28.5 (6.1)	26.8 (7.0)	30.5 (6.0)
Disease duration (years), mean (SD)	5.0 (7.0)	5.8 (7.6)	3.1 (4.9)
Number of MRI scans (n)	211	125	86
Disease activity**			
Tender joints count (n), median (IQR)	2 (0–4)	2 (0–4)	1 (0–4)
Swollen joints count (n), median (IQR)	0 (0–2)	1 (0–3)	0 (0–1)
CRP, mean (SD)	8.6 (16.9)	7.6 (11.9)	7.3 (13.6)
HAQ, median (IQR)	0.5 (0.1–0.9)	0.5 (0.1–0.9)	0.4 (0.2–0.9)
DAS28-CRP, mean (SD)	3.3 (1.3)	3.6 (1.3)	2.9 (1.2)
Therapy			
csDMARDs, n (%)			
Methotrexate	52 (46.4)	35 (45.5)	17 (48.6)
Leflunomide	4 (3.6)	2 (2.6)	2 (5.7)
Sulfasalazine	1 (0.9)	1 (1.3)	0 (0)
bDMARDs, n (%)			
TNFi	12 (10.7)	8 (10.4)	4 (11.4)
IL6i	2 (1.8)	2 (2.6)	–
IL17i	4 (3.6)	–	4 (11.4)
IL12/23i	1 (0.9)	–	1 (2.9)
Abatacept	1 (0.9)	0 (0)	1 (2.9)
Rituximab	4 (3.6)	4 (5.2)	–
tsDMARDs, n (%)			
JAKi	20 (17.9)	20 (26.0)	0 (0)
Apremilast	14 (12.5)	–	14 (40.0)
MRI**			
Total RAMRIS Score, mean (SD)	–	21.4 (3.5)	–
Erosions score	–	12.9 (3.3)	–
Osteitis score	–	4.1 (1.0)	–
Synovitis score	–	4.4 (0.7)	–
Total PsAMRIS Score, mean (SD)	–	–	7.2 (5.7)
Erosions score	–	–	1.2 (1.6)
Osteitis score	–	–	0.5 (1.8)
Synovitis score	–	–	1.4 (2.6)
Tenosynovitis score	–	–	0.4 (1.2)
Periarticular inflammation score	–	–	0.5 (1.9)
Proliferation score	–	–	5.7 (7.2)

*Disease activity and imaging parameters at the time of each MRI scan were used.

BMI, body mass index; CRP, C reactive protein; (cs/b/ts)DMARDs, conventional synthetic/biological/targeted synthetic disease-modifying anti-inflammatory drugs; DAS28, Disease Activity Score 28; HAQ, Health Assessment Questionnaire; ILi, interleukin inhibitors; JAKi, Janus kinase inhibitors; PsA, psoriatic arthritis; PsAMRIS, Psoriatic Arthritis MRI Score; RA, rheumatoid arthritis; RAMRIS, Rheumatoid Arthritis MRI Score; TNFi, tumour necrosis factor inhibitors.

For the cross-validation dataset, MRI data of 211 scans were retrieved, corresponding to a total of 14 906 ROIs. Depending on the number of landmarks per lesion and the number of available MRIs, the total number of ROIs per lesion per MRI sequence ranged from a minimum of 1297 (T1 fs CE synovitis) to a maximum of 6388 (T1 osteitis) (table 2).

**Table 2:** Number of ROIs available for each MRI sequence type per lesion training/ internal validation dataset

Score	Erosion				Osteitis				Synovitis			
	T1	T1 fs CE	T2 fs	Equal	T1	T1 fs CE	T2 fs	Equal	T1	T1 fs CE	T2 fs	Equal
Total	6377	3754	5187	3682	6388	3755	5193	3683	2141	1297	1742	1273
0	5046	3062	4126	2990	5910	3525	4816	3453	1517	1024	1280	1000
1	793	428	633	428	264	124	207	124	377	164	274	164
2	290	160	236	160	139	58	107	58	174	81	138	81
3+*	248	104	192	104	75	48	63	48	73	28	50	28

Score: Rheumatoid Arthritis MRI Score-based and Psoriatic Arthritis MRI Score-based score for erosion, osteitis and synovitis. Equal: number of ROIs for which all sequences were available.

*The 3+ category was only used for erosions. Scores for osteitis and synovitis ranged from 0 to 3.

CE, contrast enhanced; fs, fat suppressed; ROIs, region of interest.

The validation cohort included another 75 patients who contributed with 220 scans. The number of ROIs ranged from 1452 (T1 fs CE synovitis) to 4416 (T1 erosion and osteitis) (online supplemental table S1). Compared with the training/internal validation cohort, PsA (n=62) was more prevalent than RA (n=13), but the age and sex distributions were similar. MRI signs of inflammation were less pronounced in the validation scans, with a mean (SD) RAMRIS Score of 14.7 (7.5) and a mean (SD) PsAMRIS Score of 4.8 (4.6). Detailed patient characteristics of the validation group are described in online supplemental table S2.

### Automated scoring of erosions, osteitis and synovitis

The convolutional neural networks (CNNs) were trained separately for erosions, osteitis and synovitis and showed high performance as seen in table 3. To consider each class equally, independent of class imbalance, macro AUC is used as the main evaluation measure. Since the number of available ROIs is considerably different between MRI sequences, we present two evaluations: one considering all available MRI images, and a second considering only the cases where images are accessible for all MRI sequences (*seq-equal*) to avoid that the dataset itself is the reason for differences in performance between sequences. Further, we provide evaluations for voting ensembles as a combination of two or all used MRI sequences. Thereby, we combine the prediction probabilities, for example, for T1 and T2 fs, and average those to a combined probability vote.

**Table 3:** Macro AUC and balanced accuracy for all pathologies and all MRI sequences and combination of sequences during cross-validation

MRI sequence	Erosion		Osteitis		Synovitis	
	Macro AUC	Balanced accuracy	Macro AUC	Balanced accuracy	Macro AUC	Balanced accuracy
T1 fs CE	87%±4%	61%±6%	89%±2%	55%±4%	84%±3%	**64%±6%**
T1	91%±1%	**65%±2%**	85%±2%	49%±5%	81%±3%	55%±2%
T2 fs	88%±2%	61%±3%	88%±3%	**57%±5%**	78%±2%	54%±3%
T1 fs CE+T2 fs	88%±3%	62%±3%	89%±3%	55%±4%	83%±2%	58%±3%
T1+T1 fs CE	90%±2%	64%±4%	88%±2%	48%±3%	84%±2%	58%±2%
T1+T2 fs	**92%±1%**	**65%±3%**	90%±2%	51%±5%	82%±2%	55%±3%
Majority voting	**92%±1%**	**65%±3%**	**91%±2%**	52%±4%	**85%±2%**	59%±2%

Bold text highlights the best results for the prediction of each lesion score, underlined text highlights the best performing individual sequence for each lesion. Results that are both bold and underlined reflect that one sequence alone achieves the best overall performance to predict the lesion score without need of combination with other sequences.

AUC, area under the receiver operating characteristic curve; CE, contrast enhanced; fs, fat suppressed.

The automatic scoring performed equally well for either using all available MRI images or *seq-equal*. Depending on the number of MRI images available, the best individual sequence and best voting ensemble changed. Across the five fold, the algorithm reached a mean macro AUC (mean and SD) of 92%±1% for erosion, 91%±2% for osteitis and 85%±2% for synovitis using majority voting with all available MRI sequences. Regarding erosion, the best individual MRI sequence is T1 with a macro AUC of 91%. For *seq-equal*, the sequences T1 and T1 fs CE both achieve a macro AUC of 90%. For osteitis, T1 fs CE achieves the highest macro AUC of 89%. Nonetheless, T2 fs provides a higher balanced accuracy of 57%. For *seq-equal*, the voting ensemble of T1 fs CE and T2 fs sequences achieve a macro AUC of 93% compared with 92% for majority voting. In the case of synovitis, T1 fs CE achieves the highest macro AUC for an individual MRI sequence of 84%. For *seq-equal*, a combination of T1 and T1 fs CE or all three sequences achieve a macro AUC of 87%. Detailed results on the CNN evaluation of erosions, osteitis and synovitis during cross-validation in the training/internal validation dataset are provided in online supplemental tables S3–5 and S6–8 for *seq-equal*.

**Figure 2:**
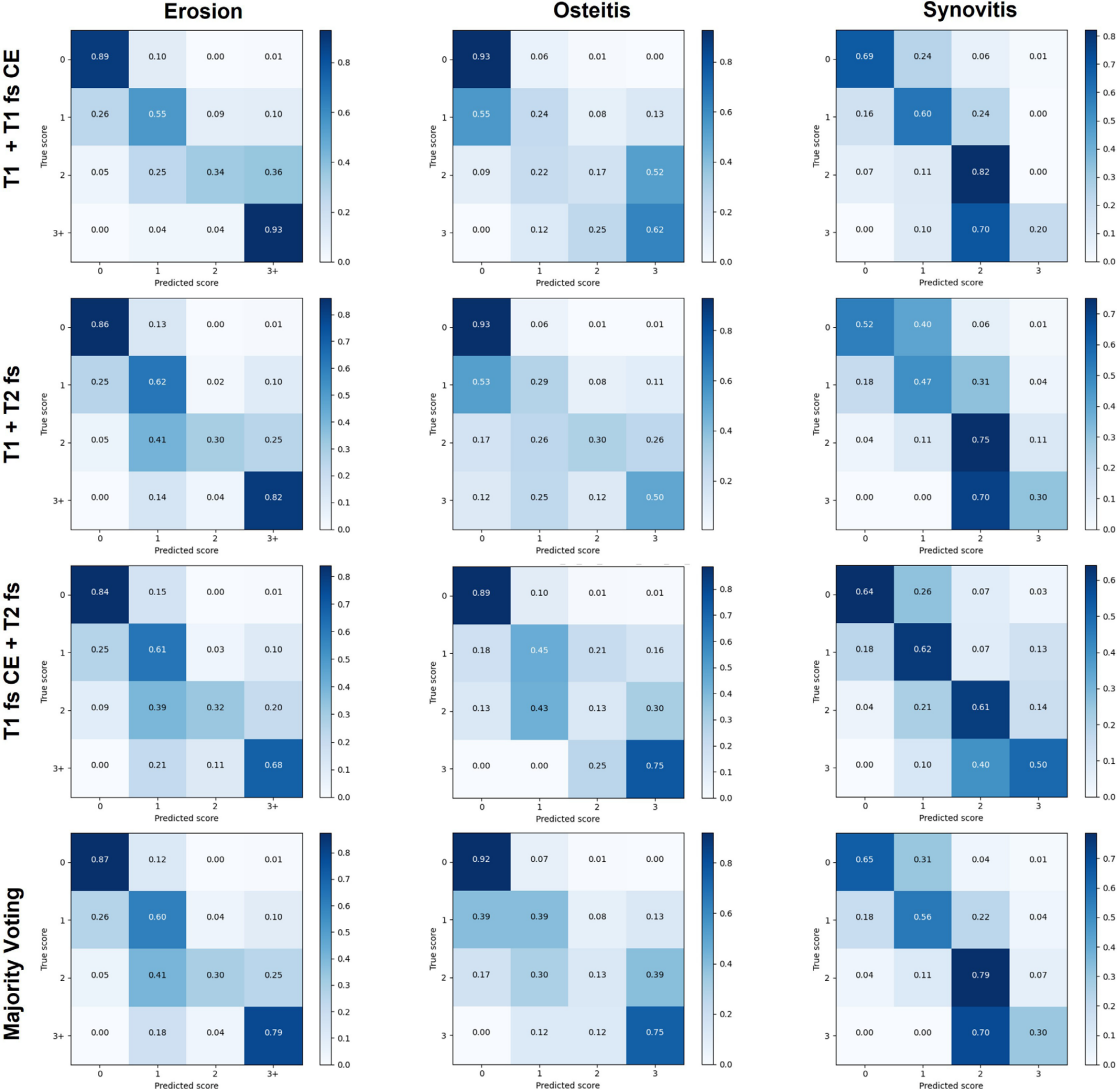
Confusion matrices for all pathologies of all combinations for all folds of cross-validation. Horizontally are the true scores, vertically the predicted scores by the network. CE, contrast enhanced; fs, fat suppressed.

The confusion matrices as shown in figure 2 provide detailed insight into correct and false predictions. For most cases, the main weight is distributed among the diagonal axis, which corresponds to correctly predicted scores. For synovitis, higher scores deviate from the diagonal indicating score underestimation.

### Scoring of pathological changes in individual patients

**Figure 3:**
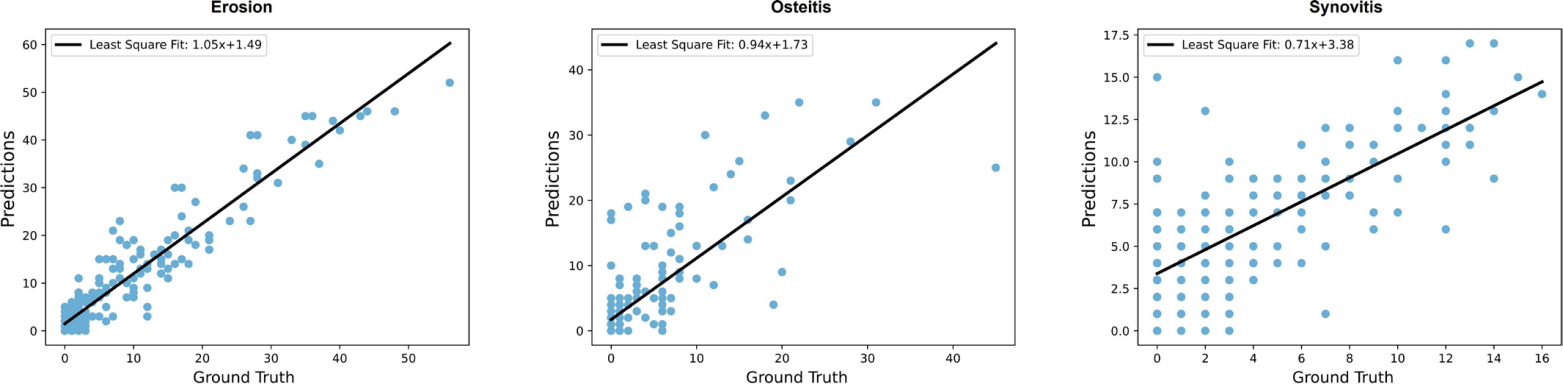
Sum of majority vote for all predictions per hand plotted against the actual sum of all joint scores rated by an expert during cross-validation.

To better access the general disease activity of a patient, the sums of the scores relative to each pathological change were combined for each hand. In figure 3, we present the correlation analysis between the CNN predictions of pathological changes and human scoring. We observed a high Spearman correlation (mean and SD) for the hand score of 90±2% for erosion, 78±8% for osteitis and 69±7% for synovitis indicating a strong agreement between the automated predictions and human scores on a patient level. For each pathological change, we performed a least-squares fit analysis, resulting in a slope of 1.05 and an intersection of 1.49 for erosion, 0.94 and 1.73 for osteitis and 0.71 and 3.37 for synovitis. These values provide insights into the relationship between the automated predictions and human scores for each specific pathological change. They indicate that the predictive capability for erosions is very high, for osteitis the predicted score is higher than the human scores and for synovitis lower scores are overestimated.

### Performance of the algorithm in the independent validation cohort

During validation, we calculated the predictions using an ensemble of the respective networks trained for cross-validation. Using a completely disjunct dataset for validation, our trained networks achieved slightly lower results compared with cross-validation. Still, we could reach a macro AUC of 80% for erosions using an ensemble of all three sequences on the individual ROIs, as depicted in table 4. Here, the best individual sequence is T1 fs CE with a macro AUC of 79%. For osteitis the best performing sequence is T1 fs CE with a macro AUC of 86%. In the case of synovitis, again the majority voting achieves the best results with a macro AUC of 77% and the best individual sequence T1 with a macro AUC of 75%. Detailed results on the CNN evaluation of erosions, osteitis and synovitis in the validation dataset are provided in online supplemental tables S9–11. Confusion matrices of all combinations of MRI sequences can be found in online supplemental figure S1. Online supplemental figure S2 shows the summed score on the full hand of each pathology, which reaches a Spearman correlation of 65%, 73% and 34% for erosions, osteitis and synovitis.

**Table 4:** Macro AUC and balanced accuracy for all pathologies and all MRI sequences and combination of sequences during independent validation

MRI sequence	Erosion		Osteitis		Synovitis	
	Macro AUC	Balanced accuracy	Macro AUC	Balanced accuracy	Macro AUC	Balanced accuracy
T1 fs CE	87.92%	** 49.66% **	**96.09%**	**53.73%**	83.67%	41.73%
T1	85.95%	46.37%	89.24%	45.86%	85.33%	40.2%
T2 fs	86.84%	39.71%	94.86%	46.75%	84.96%	**47.08%**
T1 fs CE+T2 fs	85.52%	43.11%	**95.61%**	46.14%	87.84%	43.23%
T1+T1 fs CE	**86.9%**	44.72%	92.33%	48.95%	88.07%	40.5%
T1+T2 fs	**88.13%**	43.94%	94.53%	42.96%	87.19%	40.55%
Majority voting	**87.89%**	43.56%	**95.64%**	47.26%	**89.13%**	41.17%

Bold text highlights the best results for the prediction of each lesion, underlined text highlights the best performing individual sequence. Results that are both bold and underlined reflect that one sequence alone achieves the best overall performance to predict the lesion score without need of combination with other sequences.

AUC, area under the receiver operating characteristic curve; CE, contrast enhanced; fs, fat suppressed.

### Impact of training data size on performance

**Figure 4:**
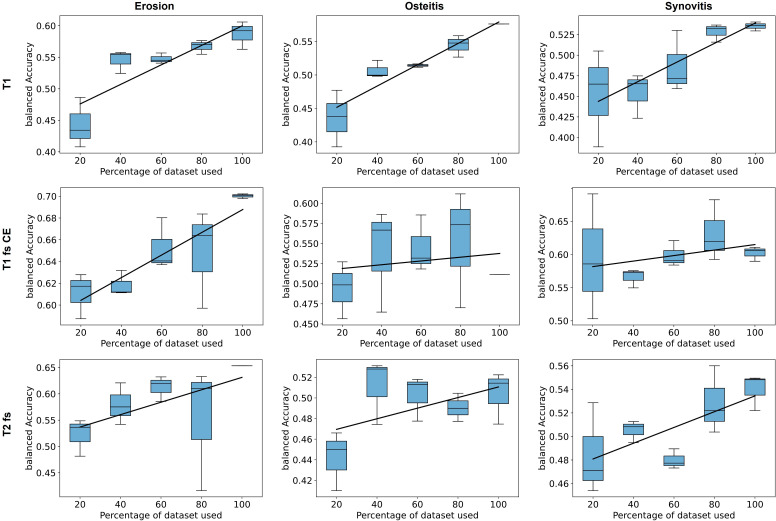
Subset analysis of all MRI sequences and pathologies with percentages of training data ranging from 20% to 100% with three repetitions while keeping the test set fixed. CE, contrast enhanced; fs, fat suppressed.

Decreasing the size of the patient cohort used for training the neural network significantly impacted the predictions for the test set. As shown in figure 4, we observed a positive correlation between the number of patients and the network’s predictive performance for each pathological change and MRI sequence. The random selection of the training data subset highlights the substantial influence of data variability within the training dataset. This impact becomes evident when assessing the scoring performance on a test set. In fact, when a random subset of images is selected for training, the obtained results might not reflect the data that is available during testing.

### Reader study

The two readers achieved a Cohen’s kappa of 89% for erosion, 86% for osteitis and 84% for synovitis. The macro AUC between the readers is 97%, 94% and 90%, for erosion, osteitis and synovitis. The balanced accuracy for each task is 93%, 94% and 89%, respectively.

## Discussion

In this study, we successfully trained and validated a CNN to automatically predict and score the severity of erosions, osteitis and synovitis in hand MRI images in patients with inflammatory arthritis.

In recent years, the interest for AI applications to musculoskeletal MRI has been growing rapidly. Yet, CNN-based and AI-based segmentation tools developed so far have been mainly focused on osteoarthritis and more specifically on the knee and shoulder joints.^31^ These CNN-based approaches yielded high diagnostic accuracy for the classification of bone and soft tissue lesions including subchondral oedema, fractures, joint effusions and ligament and tendon tears,^32^ but were not trained on smaller joints or for inflammatory lesions that are more relevant and specific for arthritis. For other forms of imaging, AI-based models for the recognition of inflammatory arthritis in the small joints of the hand and finger has already proved successful. Ahalya *et al* trained and validated a CNN that was able to classify thermal images of the hand of patients with RA against healthy controls with high accuracy,^33^ while an approach by Phatak *et al* was successfully trained to detect synovitis of the wrist and finger joints based on hand photographs.^34^ Regarding MRI of the hands, however, AI applications so far have been very limited and have been exclusively focused on the classification of different types of arthritis^18^ or of early disease against non-arthritic controls,^19^ while automatic methods for scoring arthritis severity quantitatively and based on the recognition of individual inflammatory lesions are missing.

In our MRI-based CNN model for hand arthritis, each classification network reached a high macro AUC and correlated strongly with human annotation. Compared with erosions and osteitis, for which we observed higher performances, accuracy for the prediction of synovitis was moderate. The validation of the algorithm on a further cohort of independent patients provided similar results: while correlations slightly dropped, they remained robust and consistent with the ones observed during training.

The model demonstrated high reliability to predict scores for individual joints, which allows a cumulative scoring of the whole hand. Further, we observed that in most cases the use of only one MRI sequence achieved comparable or even better performances compared with a combination of sequences. Most relevantly, T1w and T2w fs MRI sequences without contrast enhancement performed comparably to CE sequences for the detection of erosions. This is particularly relevant, as limiting the number of sequences and omitting the contrast agent would allow to substantially reduce scanning times, costs, as well as risks for the patient. However, CE still showed relevant impact for the classification of osteitis and synovitis, especially in the case where an equal amount of data for each sequence type is used. At the same time, we showed that the current results will likely benefit from increasing the number of training samples, since we do not see saturation effects when using different training set sizes. Interestingly, this observation could mean that CE sequences could potentially be substituted in the assessment of osteitis and synovitis if larger amounts of training data for T1 and T2 fs sequences are provided. This could prove more challenging for synovitis, since obtaining an equal performance for its detection on non-CE MRI would require to exclude confounding or accompanying factors being falsely recognised as synovitis.

Our results should be interpreted in the context of some limitations. First, the datasets for training and validation spawned from a single centre. Second, the model underestimated erosions with high RAMRIS scores, as it was limited to scores of 3+ and not up to 10 for erosion. However, this could be overcome by including more patients with higher disease activity and training the network with all 10 classes. Third, when comparing results across pathologies, the automated detection of osteitis and synovitis proved slightly more challenging. This could be attributed to the presence of a relatively low frequency of high scores for osteitis and synovitis and to generally lower disease activity and lower representation of RA patients in the validation dataset. Since the mean overall burden of pathology *per* single scan in the validation dataset was lower than the one used for cross-validation, the Pearson correlations on the validation dataset were lower. Nonetheless, while the classifiers occasionally generated false predictions for classes 2 and 3+ for single lesions, the correlation between CNN predictions and the human reader remained consistently high when considering the hand as a whole. Also, as observed in the reader study, there was an overall high agreement between individual human annotators. Thus, the influence of reader bias can be considered neglectable. While we observed a high inter-rater agreement, it could be interesting to integrate uncertainty based on rater agreement into the training process to improve robustness.

Regarding the study cohort, some aspects might limit the applicability of the current version of our CNN to specific groups of patients. First, patients with RA and PsA with imaging signs of secondary osteoarthritis were not removed from the analysis and no control group without osteoarthritis was present. In addition, our CNN was not tested on a non-arthritic control group. Last, PsA patients were over-represented in the external validation dataset compared with the training dataset, where RA was more prevalent, which could lead to better accuracy in RA-only cohorts compared with PsA. As such, further training of the CNN on broader cohorts of patients as well as on non-arthritic control is warranted in order to ensure broader clinical applicability. Nonetheless, by analysing our independent validation cohorts separately, despite the higher prevalence of PsA in the PSARTROS group, the algorithm did not substantially underperform in the recognition of erosions, synovitis and osteitis compared with the PARAJA group (online supplemental tables S12–17). Indeed, it is worth noting that the elementary MRI lesions tested by this CNN (ie, synovitis, osteitis, erosions) are morphologically similar in RA, PsA, spondyloarthritis and other forms of seronegative and crystal arthritis, so that this kind of CNN still retains high potential for broader generalizability, which could explain the good results obtained in our external validation.

To the best of our knowledge, our study is the first to explore the automated scoring of erosions, synovitis and osteitis using MRI data. In conclusion, our deep learning-based scoring system of inflammatory MRI changes of the hand based on RAMRIS and PsAMRIS scores shows high potential for future clinical applications. Still, translating the use of our system into the clinical workflow such as in the setting of a clinical trial requires further technical improvements and research efforts. To ensure a broader applicability of similar machine learning approaches, larger cohorts from multiple centres including different MRI devices and with an adequate representation the different diagnoses should be used for training, especially if one aims to detect disease-specific lesions such as periarticular inflammation and enthesophytes. Also, MRI-based CNNs need to be longitudinally validated on prospective data to assess their sensitivity to changes within the same patient. Last, automating the process of landmark detection would substantially improve the efficiency of training MRI-based CNNs.

If successful, this approach has the potential of enabling an accurate and unbiased automated scoring of pathological changes that could optimise patients’ follow-up and clinical decision-making. To facilitate this, we make the whole annotation pipeline for landmark detection and the classification network architecture available on request.

## Data Availability

Data are available on reasonable request.
