# Supplementary material for: Deep learning-based classification of erosion, synovitis and osteitis in hand MRI of patients with inflammatory arthritis
Source: RMD Open. 2024 Jun 17;10(2):e004273. doi: 10.1136/rmdopen-2024-004273 (PMC11184189; doi:10.1136/rmdopen-2024-004273)

Supplementary information to:

Deep Learning-based Classification of Erosion, Synovitis and Osteitis in Hand MRI of Patients with Inflammatory Arthritis

Maja Schlereth[1], Melek Yalcin-Mutlu[2,3], Jonas Utz[1], Sara Bayat[2,3], Tobias Heimann[4], Jingna Qiu[1], Chris Ehring[5], Chang Liu[6], Michael Uder[5], Arnd Kleyer[2,3,7], David Simon[2,3,7], Frank Roemer[5,8], Georg Schett[2,3], Katharina Breininger[1*], Filippo Fagni[2,3*]

[1]Department Artificial Intelligence in Biomedical Engineering, Friedrich-Alexander-Universität Erlangen-Nürnberg, Erlangen, Germany

[2]Department of Internal Medicine 3, Friedrich-Alexander-University Erlangen-Nürnberg (FAU) and, Universitätsklinikum Erlangen, Erlangen, Germany

[3]Deutsches Zentrum Immuntherapie (DZI), Friedrich-Alexander-University Erlangen-Nürnberg (FAU) and Universitätsklinikum Erlangen, Erlangen, Germany

[4]Digital Technology and Innovation, Siemens Healthcare GmbH, Erlangen, Germany

[5]Institute of Radiology, Friedrich-Alexander-University Erlangen-Nürnberg (FAU) and Universitätsklinikum Erlangen, Erlangen, Germany

[6]Pattern Recognition Lab, Department of Computer Science, Friedrich-Alexander-Universität Erlangen-Nürnberg, Erlangen, Germany

[7]Department of Rheumatology and Clinical Immunology, Charité - Universitätsmedizin Berlin

[8]Department of Radiology, Boston University School of Medicine, Boston, MA, USA

*Contributed equally

Supplementary methods

The region of interests (ROIs) included in the RAMRIS and in PsAMRIS scoring systems were utilized for our analysis. Namely for synovitis: radioulnar joint, radiocarpal joint, intercarpal joint, metacarpophalangeal joints (MCPs), proximal (PIPs) and distal interphalangeal joints (DIPs); for erosion-edema: distal ulna, distal radius, carpal bones, metacarpal bases, proximal and distal MCPs, PIPs, and DIPs.

Supplementary tables and figures

Table S1 – Number of ROIs available for each MRI sequence type per lesion pattern for validation.

Score	Erosion			Osteitis			Synovitis		
	**T1**	**T1 fs CE**	**T2 fs**	**T1**	**T1 fs CE**	**T2 fs**	**T1**	**T1 fs CE**	**T2 fs**
total	4416	2904	3504	4416	2904	3504	2208	1452	1752
0	3962	2615	3149	4300	2806	3401	1930	1279	1544
1	280	173	213	40	30	32	211	122	154
2	65	38	49	20	16	16	39	23	27
3+*	56	29	42	5	3	5	4	4	3

Score: RAMRIS- and PsAMRIS-based score for erosion, osteitis, and synovitis. Equal: Number of ROIs for which all sequences were available. *The 3+ category was only used for erosions. Scores for osteitis and synovitis ranged from 0 to 3.

Table S2 – Patient clinical and imaging characteristics of the validation dataset.

	**Total**	**RA**	**PsA**
**N**	75	13	62
**Demographics**			
Female sex, n (%)	34 (45.3)	7 (53.8)	27 (43.5)
Age (years), mean (SD)	56.9 (10.6)	57.7 (9.4)	56.4 (10.4)
BMI (kg/m^2), mean (SD)	30.1 (5.4)	28.3 (4.0)	30.3 (5.5)
Disease duration (years), mean (SD)	7.0 (9.4)	5.8 (7.6)	7.2 (8.7)
Number of MRI scans (n)	220	22	198
**Disease activity***			
Tender joints count (n), median (IQR)	3 (0-6)	5 (2-11)	2 (0-5)
Swollen joints count (n), median (IQR)	0 (0-1)	0 (0-3)	0 (0-1)
CRP, mean (SD)	6.1 (11.0)	4.2 (1.6)	6.6 (11.6)
HAQ, median (IQR)	0.6 (0.2-1.0)	0.5 (0.1-1.1)	0.6 (0.3-1.0)
DAS28-CRP, mean (SD)	3.3 (1.4)	4.2 (1.7)	3.2 (1.3)
**Therapy**			
csDMARDs, n (%)			
Methotrexate	39 (52.0)	9 (69.2)	30 (48.4)
Leflunomide	3 (4.0)	2 (15.4)	1 (1.6)
Sulfasalazine	3 (4.0)	0 (0)	3 (4.8)
bDMARDs, n (%)			
TNFi	14 (18.7)	6 (46.2)	8 (12.9)
IL6i	0 (0)	0 (0)	-
IL17i	22 (29.3)	-	22 (35.5)
IL12/23i	3 (4.0)	-	3 (4.8)
Abatacept	0 (0)	0 (0)	0 (0)
Rituximab	0 (0)	0 (0)	-
tsDMARDs, n (%)			
JAKi	55 (73.3)	13 (100.0)	42 (67.7)
Apremilast,	0 (0)	-	0 (0)
**MR Imaging***			
Total RAMRIS score, mean (SD)	-	14.7 (7.5)	-
Erosions score	-	7.3 (3.5)	-
Osteitis score	-	1.2 (1.8)	-
Synovitis score	-	2.6 (2.4)	-

Total PsAMRIS score, mean (SD)	-	-	4.8 (4.6)
Erosions score	-	-	1.4 (1.8)
Osteitis score	-	-	0.2 (0.7)
Synovitis score	-	-	0.9 (1.4)
Tenosynovitis score	-	-	0.7 (1.8)
Periarticular inflammation score	-	-	0.4 (0.8)
Proliferation score	-	-	1.0 (1.6)

*Disease activity and imaging parameters at the time of each MRI scan were used.
SD: standard deviation; IQR: interquartile range; RA: rheumatoid arthritis; PsA: psoriatic arthritis; BMI: body mass index; MRI: magnetic resonance imaging; CRP: C-reactive protein; HAQ: health assessment questionnaire; DAS28: disease activity score 28; NSAID: non-steroidal anti-inflammatory drugs; (cs/b/ts)DMARDs: conventional synthetic/biological/targeted synthetic disease-modifying anti-inflammatory drugs; TNFi: tumor necrosis factor inhibitors; ILi: interleukin inhibitors; JAKi: Janus kinase inhibitors. RAMRIS: RA MRI score; PsAMRIS: PsA MRI score.

Table S3 – Detailed evaluation for erosions of each MRI sequence and combination of sequences during cross-validation. Depicted are weighted AUC, balanced accuracy, macro AUC, weighed and macro PR-AUC.

Erosions Cross-Validation	Weighted AUC	Balanced Accuracy	Macro AUC	Weighted PR-AUC	Macro PR-AUC
T1 fs CE	89% ± 5%	61% ± 6%	87% ± 4%	86% ± 3%	54% ± 5%
T1	92% ± 2%	**65% ± 2%**	91% ± 1%	87% ± 1%	60% ± 2%
T2 fs	90% ± 3%	61% ± 3%	88% ± 2%	85% ± 2%	55% ± 5%
T1 fs CE + T2 fs	90% ± 3%	62% ± 3%	88% ± 3%	86% ± 2%	56% ± 4%
T1 + T1 fs CE	92% ± 2%	64% ± 4%	90% ± 2%	87% ± 1%	60% ± 2%
T1 + T2 fs	**93% ± 2%**	**65% ± 3%**	**92% ± 1%**	**88% ± 2%**	**63% ± 4%**
Majority Voting	**93% ± 2%**	**65% ± 3%**	**92% ± 1%**	**88% ± 1%**	**63% ± 3%**

Table S4 – Detailed evaluation for osteitis of each MRI sequence and combination of sequences during cross-validation. Depicted are weighted AUC, balanced accuracy, macro AUC, weighed and macro PR-AUC.

Osteitis Cross-Validation	Weighted AUC	Balanced Accuracy	Macro AUC	Weighted PR-AUC	Macro PR-AUC
T1 fs CE	91% ± 3%	55% ± 4%	89% ± 2%	**95% ± 1%**	42% ± 6%
T1	88% ± 3%	49% ± 5%	85% ± 2%	93% ± 1%	39% ± 5%
T2 fs	91% ± 3%	**57% ± 5%**	88% ± 3%	94% ± 1%	45% ± 6%
T1 fs CE + T2 fs	91% ± 3%	55% ± 4%	89% ± 3%	94% ± 1%	45% ± 5%
T1 + T1 fs CE	91% ± 2%	48% ± 3%	88% ± 2%	94% ± 1%	42% ± 7%
T1 + T2 fs	**93% ± 1%**	51% ± 5%	90% ± 2%	94% ± 1%	48% ± 4%
Majority Voting	**93% ± 2%**	52% ± 4%	**91% ± 2%**	94% ± 1%	**49% ± 3%**

Table S5 – Detailed evaluation for synovitis of each MRI sequence and combination of sequences during cross-validation. Depicted are weighted AUC, balanced accuracy, macro AUC, weighed and macro PR-AUC.

Synovitis Cross-Validation	Weighted AUC	Balanced Accuracy	Macro AUC	Weighted PR-AUC	Macro PR-AUC
T1 fs CE	85% ± 5%	**64% ± 6%**	84% ± 3%	**83% ± 4%**	57% ± 5%
T1	81% ± 3%	55% ± 2%	81% ± 3%	76% ± 3%	49% ± 3%
T2 fs	80% ± 4%	54% ± 3%	78% ± 2%	75% ± 4%	46% ± 3%
T1 fs CE + T2 fs	84% ± 4%	58% ± 3%	83% ± 2%	79% ± 3%	54% ± 4%
T1 + T1 fs CE	84% ± 3%	58% ± 2%	84% ± 2%	79% ± 3%	54% ± 3%
T1 + T2 fs	83% ± 3%	55% ± 3%	82% ± 2%	78% ± 0%	54% ± 3%
Majority Voting	**86% ± 2%**	59% ± 2%	**85% ± 2%**	80% ± 2%	**58% ± 3%**

Table S6 – Detailed evaluation for erosions of each MRI sequence and combination of sequences during cross-validation when the same number of available data is used for each sequence (*seq equal*). Depicted are weighted AUC, balanced accuracy, macro AUC, weighed and macro PR-AUC.

Erosions equal	Weighted AUC	Balanced Accuracy	Macro AUC	Weighted PR-AUC	Macro PR-AUC
T1 fs CE	92% ± 3%	64% ± 6%	90% ± 3%	88% ± 2%	60% ± 4%
T1	91% ± 4%	68% ± 7%	90% ± 1%	88% ± 2%	60% ± 4%
T2 fs	89% ± 4%	63% ± 4%	87% ± 3%	86% ± 2%	55% ± 1%
T1 fs CE + T2 fs	93% ± 3%	63% ± 7%	92% ± 2%	89% ± 2%	63% ± 4%
T1 + T1 fs CE	**94% ± 3%**	67% ± 4%	93% ± 2%	**90% ± 2%**	67% ± 5%
T1 + T2 fs	93% ± 3%	**70% ± 5%**	92% ± 2%	89% ± 3%	67% ± 6%
Majority Voting	**94% ± 2%**	69% ± 6%	**94% ± 1%**	**90% ± 2%**	**70% ± 6%**

Table S7– Detailed evaluation for osteitis of each MRI sequence and combination of sequences during cross-validation when the same number of available data is used for each sequence (*seq equal*). Depicted are weighted AUC, balanced accuracy, macro AUC, weighed and macro PR-AUC.

Osteitis equal	Weighted AUC	Balanced Accuracy	Macro AUC	Weighted PR-AUC	Macro PR-AUC
T1 fs CE	**93% ± 3%**	53% ± 3%	92% ± 2%	**95% ± 1%**	44% ± 9%
T1	87% ± 5%	55% ± 5%	84% ± 5%	94% ± 2%	38% ± 7%
T2 fs	91% ± 4%	55% ± 4%	87% ± 2%	**95% ± 1%**	42% ± 6%
T1 fs CE + T2 fs	**93% ± 2%**	**56% ± 5%**	**93% ± 2%**	**95% ± 1%**	46% ± 7%
T1 + T1 fs CE	92% ± 3%	54% ± 2%	90% ± 5%	**95% ± 1%**	46% ± 10%
T1 + T2 fs	92% ± 4%	53% ± 2%	89% ± 2%	**95% ± 1%**	45% ± 10%
Majority Voting	**93% ± 3%**	**56% ± 7%**	92% ± 2%	**95% ± 1%**	**48% ± 8%**

Table S8 – Detailed evaluation for synovitis of each MRI sequence and combination of sequences during cross-validation when the same number of available data is used for each sequence (*seq equal*). Depicted are weighted AUC, balanced accuracy, macro AUC, weighed and macro PR-AUC.

Synovitis equal	Weighted AUC	Balanced Accuracy	Macro AUC	Weighted PR-AUC	Macro PR-AUC
T1 fs CE	86% ± 5%	**64% ± 10%**	85% ± 4%	**84% ± 4%**	**57% ± 8%**
T1	80% ± 5%	52% ± 6%	77% ± 3%	80% ± 3%	45% ± 11%
T2 fs	81% ± 5%	54% ± 5%	79% ± 4%	80% ± 4%	45% ± 1%
T1 fs CE + T2 fs	**87% ± 4%**	60% ± 6%	86% ± 3%	83% ± 3%	54% ± 3%
T1 + T1 fs CE	**87% ± 4%**	54% ± 4%	**87% ± 2%**	83% ± 3%	53% ± 7%
T1 + T2 fs	83% ± 4%	51% ± 8%	82% ± 2%	81% ± 4%	48% ± 9%
Majority Voting	**87% ± 4%**	54% ± 6%	**87% ± 2%**	83% ± 3%	55% ± 6%

Table S9 – Detailed evaluation for erosions of each MRI sequence and combination of sequences during independent validation. Depicted are weighted AUC, balanced accuracy, macro AUC, weighed and macro PR-AUC.

Erosions Validation Cohort	Weighted AUC	Balanced Accuracy	Macro AUC	Weighted PR-AUC	Macro PR-AUC
T1 fs CE	87.92%	**49.66%**	76.97%	92.27%	46.0%
T1	85.95%	46.37%	76.34%	92.06%	48.2%
T2 fs	86.84%	39.71%	74.26%	91.5%	42.84%
T1 fs CE + T2 fs	85.52%	43.11%	73.98%	91.49%	44.01%
T1 + T1 fs CE	**86.9%**	44.72%	**77.02%**	**92.24%**	**49.24%**
T1 + T2 fs	**88.13%**	43.94%	77.27%	**92.59%**	48.35%
Majority Voting	**87.89%**	43.56%	**77.13%**	**92.37%**	48.5%

Table S10 – Detailed evaluation for osteitis of each MRI sequence and combination of sequences during independent validation. Depicted are weighted AUC, balanced accuracy, macro AUC, weighed and macro PR-AUC.

Osteitis Validation Cohort	Weighted AUC	Balanced Accuracy	Macro AUC	Weighted PR-AUC	Macro PR-AUC
T1 fs CE	**96.09%**	**53.73%**	**81.9%**	98.71%	**50.87%**
T1	89.24%	45.86%	75.44%	98.65%	47.16%
T2 fs	94.86%	46.75%	79.1%	98.71%	42.56%
T1 fs CE + T2 fs	**95.61%**	46.14%	80.05%	98.69%	45.46%
T1 + T1 fs CE	92.33%	48.95%	77.85%	98.76%	**50.26%**
T1 + T2 fs	94.53%	42.96%	79.06%	98.73%	46.28%
Majority Voting	**95.64%**	47.26%	80.47%	98.79%	48.39%

Table S11 – Detailed evaluation for synovitis of each MRI sequence and combination of sequences during independent validation. Depicted are weighted AUC, balanced accuracy, macro AUC, weighed and macro PR-AUC.

Synovitis Validation Cohort	Weighted AUC	Balanced Accuracy	Macro AUC	Weighted PR-AUC	Macro PR-AUC
T1 fs CE	83.67%	41.73%	69.4%	**90.71%**	**38.44%**
T1	85.33%	40.2%	75.78%	88.49%	37.0%
T2 fs	84.96%	**47.08%**	76.43%	88.03%	36.46%
T1 fs CE + T2 fs	87.84%	43.23%	76.12%	**89.93%**	**39.53%**
T1 + T1 fs CE	88.07%	40.5%	75.99%	**89.73%**	38.93%
T1 + T2 fs	87.19%	40.55%	77.97%	88.41%	38.26%
Majority Voting	**89.13%**	41.17%	**78.42%**	**89.58%**	40.04%

Table S12 – Detailed evaluation for erosion of each MRI sequence and combination of sequences during independent validation of the PARAJA cohort. Depicted are weighted AUC, balanced accuracy, macro AUC, weighed and macro PR-AUC.

Erosion PARAJA Cohort	Weighted AUC	Balanced Accuracy	Macro AUC	Weighted PR-AUC	Macro PR-AUC
T1 fs CE	91.78%	53.88%	79.62%	94.38%	48.01%
T1	92.24%	51.34%	81.14%	95.17%	49.78%
T2 fs	91.88%	40.52%	76.05%	94.85%	43.25%
T1 fs CE + T2 fs	92.93%	47.36%	78.93%	94.84%	45.95%
T1 + T1 fs CE	94.04%	48.44%	82.88%	95.43%	54.06%
T1 + T2 fs	93.68%	49.01%	81.15%	95.17%	49.03%
Majority Voting	94.11%	49.59%	82.19%	95.05%	50.7%

Table S13 – Detailed evaluation for osteitis of each MRI sequence and combination of sequences during independent validation of the PARAJA cohort. Depicted are weighted AUC, balanced accuracy, macro AUC, weighed and macro PR-AUC.

Osteitis PARAJA Cohort	Weighted AUC	Balanced Accuracy	Macro AUC	Weighted PR-AUC	Macro PR-AUC
T1 fs CE	94.4%	54.88%	82.69%	98.67%	37.17%
T1	92.4%	42.67%	77.23%	98.87%	33.42%
T2 fs	96.08%	35.7%	70.11%	98.84%	31.96%
T1 fs CE + T2 fs	96.43%	38.59%	71.88%	98.81%	35.74%
T1 + T1 fs CE	95.44%	44.36%	80.17%	98.95%	36.19%
T1 + T2 fs	95.01%	32.82%	64.13%	98.83%	34.19%
Majority Voting	96.67%	36.27%	66.68%	98.9%	37.02%

Table S14 – Detailed evaluation for synovitis of each MRI sequence and combination of sequences during independent validation of the PARAJA cohort. Depicted are weighted AUC, balanced accuracy, macro AUC, weighed and macro PR-AUC.

Synovitis PARAJA Cohort	Weighted AUC	Balanced Accuracy	Macro AUC	Weighted PR-AUC	Macro PR-AUC
T1 fs CE	90.84%	78.01%	93.76%	93.47%	55.7%
T1	86.51%	52.88%	83.9%	92.37%	30.74%
T2 fs	81.09%	50.43%	75.63%	92.31%	33.64%
T1 fs CE + T2 fs	88.0%	63.5%	85.44%	93.28%	45.22%
T1 + T1 fs CE	89.9%	65.41%	88.37%	93.26%	42.51%
T1 + T2 fs	85.14%	55.06%	80.55%	92.1%	32.4%
Majority Voting	88.63%	63.94%	85.06%	92.84%	39.18%

Table S15 – Detailed evaluation for erosion of each MRI sequence and combination of sequences during independent validation of the PSARTROS cohort. Depicted are weighted AUC, balanced accuracy, macro AUC, weighed and macro PR-AUC.

Erosion PSARTROS Cohort	Weighted AUC	Balanced Accuracy	Macro AUC	Weighted PR-AUC	Macro PR-AUC
T1 fs CE	89.72%	47.19%	78.48%	91.49%	47.06%
T1	88.19%	44.2%	76.42%	89.97%	49.08%
T2 fs	89.53%	41.43%	76.1%	90.28%	46.65%
T1 fs CE + T2 fs	88.33%	42.53%	75.96%	90.22%	46.73%
T1 + T1 fs CE	89.09%	43.27%	77.44%	90.65%	49.95%
T1 + T2 fs	90.67%	41.63%	77.92%	91.28%	50.37%
Majority Voting	90.25%	40.16%	78.0%	91.23%	50.79%

Table S16 – Detailed evaluation for osteitis of each MRI sequence and combination of sequences during independent validation of the PSARTROS cohort. Depicted are weighted AUC, balanced accuracy, macro AUC, weighed and macro PR-AUC.

Osteitis PSARTROS Cohort	Weighted AUC	Balanced Accuracy	Macro AUC	Weighted PR-AUC	Macro PR-AUC
T1 fs CE	98.18%	47.67%	89.83%	99.04%	53.66%
T1	85.49%	57.07%	76.78%	98.74%	50.46%
T2 fs	93.39%	46.62%	78.43%	98.9%	50.51%
T1 fs CE + T2 fs	95.5%	43.19%	83.92%	98.96%	51.51%
T1 + T1 fs CE	94.59%	48.34%	88.23%	98.87%	52.19%
T1 + T2 fs	92.79%	43.32%	83.12%	98.99%	53.1%
Majority Voting	95.25%	43.34%	89.61%	99.01%	53.59%

Table S17 – Detailed evaluation for synovitis of each MRI sequence and combination of sequences during independent validation of the PSARTROS cohort. Depicted are weighted AUC, balanced accuracy, macro AUC, weighed and macro PR-AUC.

Synovitis PSARTROS Cohort	Weighted AUC	Balanced Accuracy	Macro AUC	Weighted PR-AUC	Macro PR-AUC
T1 fs CE	77.63%	41.37%	61.86%	88.39%	40.12%
T1	83.97%	40.25%	74.82%	84.72%	49.71%
T2 fs	84.0%	40.41%	74.61%	84.54%	36.85%
T1 fs CE + T2 fs	85.42%	42.66%	73.71%	87.29%	39.84%
T1 + T1 fs CE	86.79%	37.59%	75.85%	86.88%	44.92%
T1 + T2 fs	86.02%	40.12%	76.34%	85.7%	50.33%
Majority Voting	87.17%	39.52%	76.7%	86.96%	43.46%

Table S18 – Macro AUC and balanced accuracy for all pathologies and all MRI sequences and combination of sequences during cross-validation using the Swin transformer.

MRI Sequence	Erosion		Osteitis		Synovitis	
	**Macro AUC**	**Balanced Accuracy**	**Macro AUC**	**Balanced Accuracy**	**Macro AUC**	**Balanced Accuracy**
T1 fs CE	84% ± 3%	67% ± 6%	77% ± 4%	58% ± 4%	85% ± 7%	63% ± 10%
T1	74% ± 11%	53% ± 15%	67% ± 7%	46% ± 7%	59% ± 4%	32% ± 4%
T2 fs	77% ± 7%	58% ± 8%	75% ± 2%	55% ± 2%	79% ± 3%	53% ± 3%
T1 fs CE + T2 fs	81% ± 3%	61% ± 5%	74% ± 2%	55% ± 2%	80% ± 2%	56% ± 2%
T1 + T1 fs CE	78% ± 6%	58% ± 10%	69% ± 5%	49% ± 6%	70% ± 3%	42% ± 4%
T1 + T2 fs	77% ± 5%	55% ± 8%	71% ± 3%	50% ± 5%	68% ± 3%	41% ± 3%
Majority Voting	79% ± 4%	58% ± 6%	71% ± 3%	51% ± 4%	72% ± 1%	45% ± 2%

Bold text highlights the best results for the prediction of each lesion score, underlined text highlights the best performing individual sequence for each lesion. Results that are both bold and underlined reflect that one sequence alone achieves the best overall performance to predict the lesion score without need of combination with other sequences. In one fold of Syonvitis T1 occurred training instabilities due to the network architecture. Hence, we stopped training after 50 epochs in this case.

Figure S1 – Confusion Matrices for all pathologies of all combinations of MRI sequences for the independent validation. Horizontally are the true scores, vertically the predicted scores by the network.

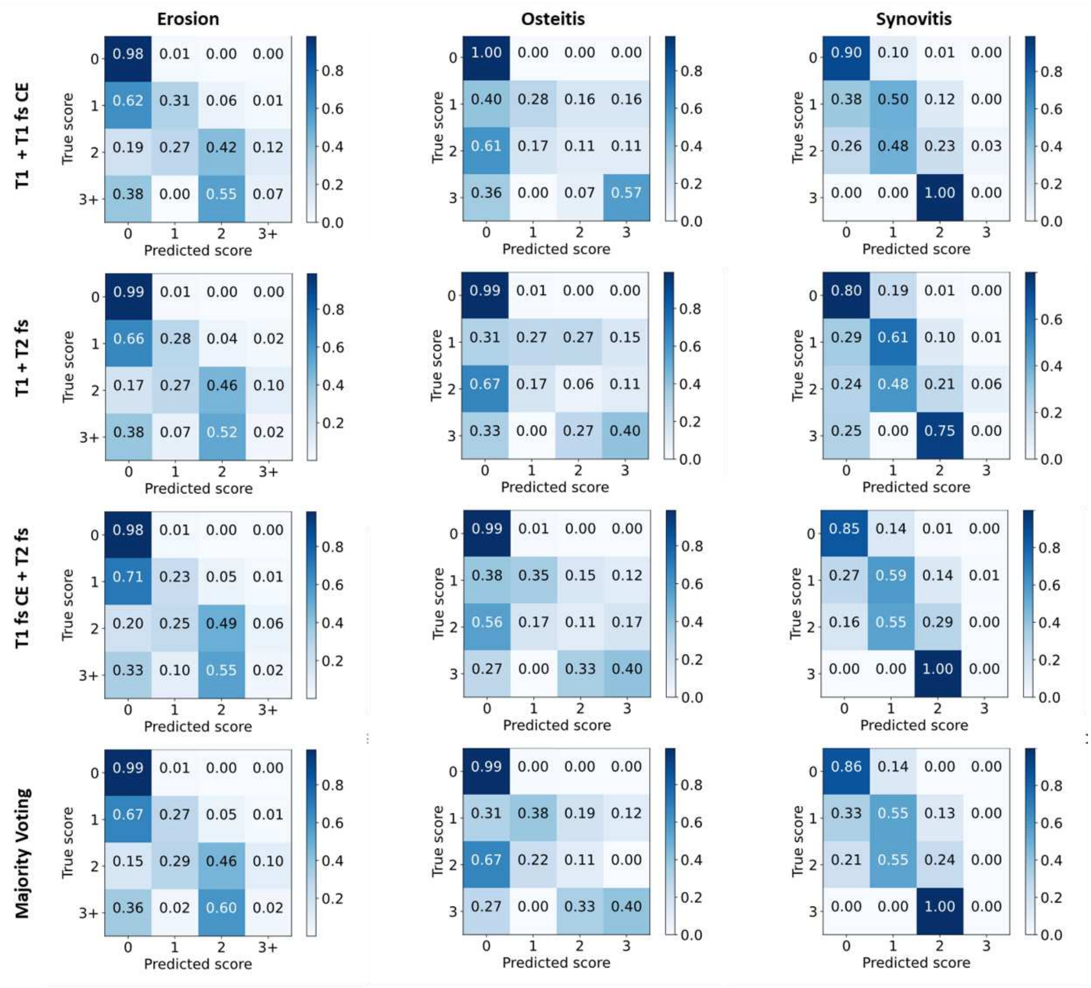

Figure S2 - Sum of majority vote for all predictions per hand plotted against the actual sum of all joint scores rated by an expert for the validation dataset.

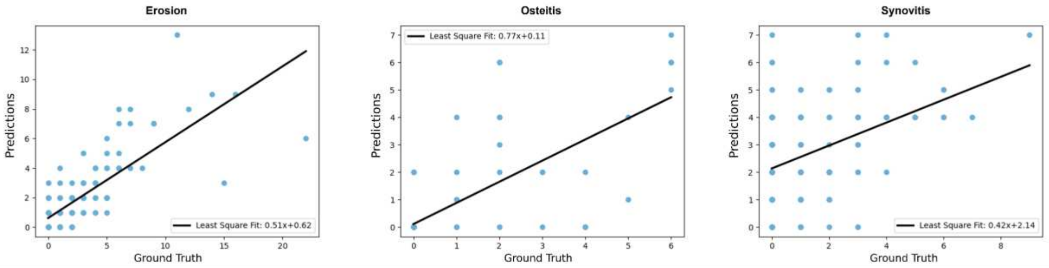

Figure S3 – Study design flow chart of training/ internal validation and external validation dataset selection and analysis.

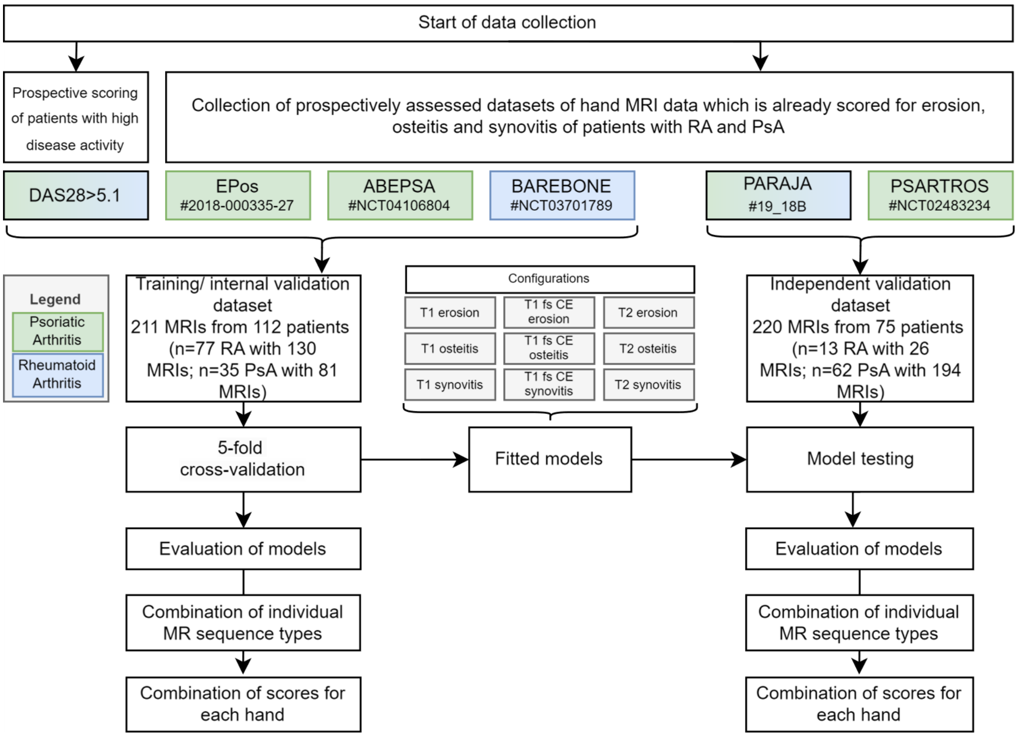

Supplement: Supplementary data [file rmdopen-2024-004273supp001.pdf]
